# Insulated *piggyBac *vectors for insect transgenesis

**DOI:** 10.1186/1472-6750-6-27

**Published:** 2006-06-16

**Authors:** Abhimanyu Sarkar, Asela Atapattu, Esther J Belikoff, Jörg C Heinrich, Xuelei Li, Carsten Horn, Ernst A Wimmer, Maxwell J Scott

**Affiliations:** 1 Centre for Functional Genomics, Institute of Molecular BioSciences, Massey University, Private Bag 11222, Palmerston North, New Zealand; 2 Lehrstuhl für Genetik, Universität Bayreuth, Universitätsstraße 30 NW1, 95447 Bayreuth, Germany

## Abstract

**Background:**

Germ-line transformation of insects is now a widely used method for analyzing gene function and for the development of genetically modified strains suitable for pest control programs. The most widely used transposable element for the germ-line transformation of insects is *piggyBac*. The site of integration of the transgene can influence gene expression due to the effects of nearby transcription enhancers or silent heterochromatic regions. Position effects can be minimized by flanking a transgene with insulator elements. The scs/scs' and *gypsy *insulators from *Drosophila melanogaster *as well as the chicken β-*globin *HS4 insulator function in both Drosophila and mammalian cells.

**Results:**

To minimize position effects we have created a set of *piggyBac *transformation vectors that contain either the scs/scs', *gypsy *or chicken β-*globin *HS4 insulators. The vectors contain either fluorescent protein or eye color marker genes and have been successfully used for germ-line transformation of *Drosophila melanogaster*. A set of the scs/scs' vectors contains the coral reef fluorescent protein marker genes AmCyan, ZsGreen and DsRed that have not been optimized for translation in human cells. These marker genes are controlled by a combined GMR-3xP3 enhancer/promoter that gives particularly strong expression in the eyes. This is also the first report of the use of the ZsGreen and AmCyan reef fluorescent proteins as transformation markers in insects.

**Conclusion:**

The insulated *piggyBac *vectors should protect transgenes against position effects and thus facilitate fine control of gene expression in a wide spectrum of insect species. These vectors may also be used for transgenesis in other invertebrate species.

## Background

Transposon-mediated germ-line transformation of insects is a powerful technique for investigating gene function [1] and is also being used for the development of insect strains suitable for area-wide control programs [2,3]. *piggyBac *is a type II (short inverted repeat DNA) transposon from the cabbage looper moth *Trichoplusia ni *[4]. Transposons related to *piggyBac *are found in the genomes of almost all eukaryotes [5] and *piggyBac *has recently been shown to be able to transpose in mammalian (human and mouse) cells as well as in the mouse germline [6]. Vectors based on the *piggyBac *element have been used for germ-line transformation of a wide range of insect species including coleoptera, dipteran, hymenopteran and lepidopteran species [7]. An analysis of 29,000 insertion events into the *Drosophila *genome found that integration was non-random but had a broader distribution than found with the *P *element [8]. In particular *piggyBac *did not show the bias for 5' regulatory regions seen with the *P *element. The site of integration, however, can influence transgene expression. Integration near to a tissue-specific transcription enhancer can lead to transgene expression in tissues and/or stages other than intended [9]. Additionally, integration into a heterochromatic region can lead to low levels of transgene expression [10]. These position effects are particularly problematic for regulated gene expression systems such as those based on the tetracycline-dependent transactivator (tTA) [11-13] and in instances where high levels of transgene expression are required. To be effective such systems need a low level of gene expression in the absence of inducer but high levels in its presence.

Position effects can be minimized by bracketing the transgene with insulator elements [14]. Insulators can both block the unwanted effects of a transcription enhancer and also minimize the effects of heterochromatin. In *Drosophila *the most widely used insulators are the specialized chromatin structures scs and scs' [15], part of the *gypsy *transposon [16] and the chicken β-*globin *5' HS4 element [17]. These insulators appear to achieve their effects by diverse mechanisms. The chicken β-*globin *HS4 insulator contains multiple elements. The enhancer-blocking element contains a binding site for the CCCTC binding factor CTCF [18]. The anti-silencer function of chicken β*-globin *HS4 likely recruits histone-modifying enzymes as a high level of histone acetylation and methylation of histone H3 at lysine 4 are localized at the insulator [19]. It is proposed that these histone modifications counteract the spreading of a compact heterochromatic structure. The scs and scs' insulators comprise a set of divergently transcribed promoters [20]. They may also function by recruiting histone modifying enzymes. The *gypsy *insulator may function by establishing loop chromatin domains with the paired insulators at the base of the loop [21].

Fluorescent proteins have been widely used to identify transgenic insects [22]. Apart from DsRed, however, the reef coral fluorescent proteins have not been used in insects although they function well as transformation markers in plants [23]. Here we report the development and evaluation of *piggyBac *vectors containing the scs/scs', *gypsy *and chicken β-*globin *HS4 insulators, some of which carry reef coral fluorescent proteins as markers.

## Results and discussion

### *piggyBac *vectors containing the scs/scs' insulator elements

**Figure 1:**
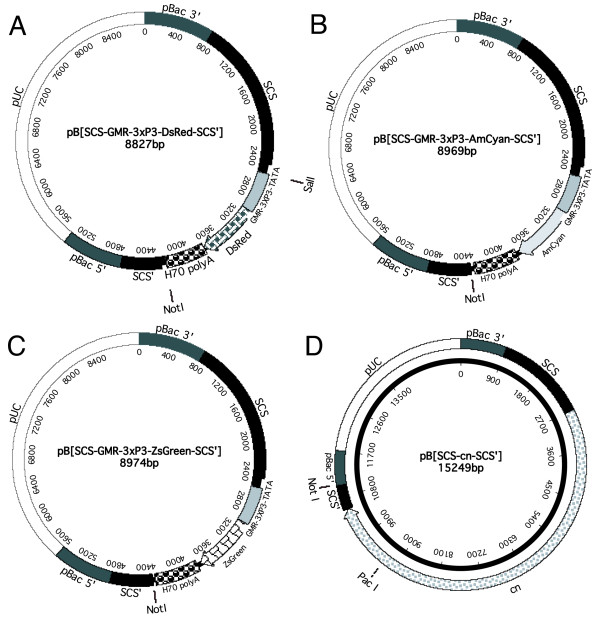
***piggyBac *vectors containing the scs/scs' insulator elements**. The vectors contain paired scs/scs' insulators flanking the DsRed (A), AmCyan (B), ZsGreen (C) and *cinnabar *(*cn*) (D) marker genes. The location of unique restriction endonuclease cloning sites are shown. The vectors have 690 bp of 5' terminal *piggyBac *sequence and either 769 bp (A-C) or 907 bp (D) of 3' terminal sequence.

To effectively minimize position effects the transgene must be bracketed by insulator elements. In this way the insulators can both block transcription enhancers and heterochromatin spreading. We have constructed four *piggyBac *vectors containing the *Drosophila *scs and scs' insulators (Figure 1). In all of these vectors the insulators flank the marker gene. Thus both the transgene and the marker gene should be protected from position effects.

**Figure 2:**
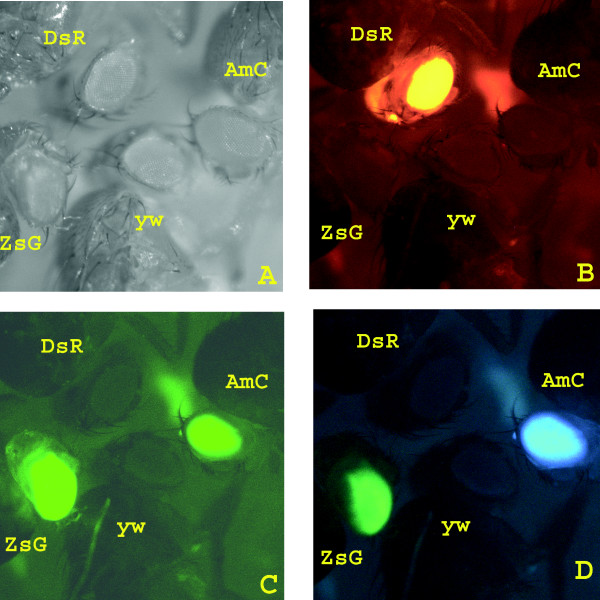
**GMR-3xP3-reef fluorescent protein marker gene expression in transgenic *Drosophila***. GMR-3xP3-DsRed, GMR-3xP3-ZsGreen and GMR-3xP3-AmCyan marker gene expression in a *yellow white *(*y w*) background observed with white light (A) or with red (B), green (C) or cyan (D) filter sets.

Three of the vectors contain reef fluorescent protein marker genes controlled by a promoter that has multiple binding sites for both the PAX6 (3xP3) and GLASS (GMR) transcription factors (Figure 2A–C). These marker genes are similar in principle to those developed previously that utilized the 3xP3 promoter [22]. However, the addition of the GMR sequence would be predicted to increase marker gene expression in the eye [24] and may be more active in some non-drosophilid species. The reef coral fluorescent proteins are available either optimized for translation in human cells or not. Codon optimization of GFP led to a significant increase in the level of GFP expression in human cells [25]. However, in some important insect pest species such as the medfly *Ceratitis capitata *and the Australian sheep blowfly *Lucilia cuprina *the protein coding genes have a very different codon bias than humans [26]. Since codon bias can significantly affect mRNA translation efficiency in insects [27], the vectors carry reef fluorescent protein genes that have not been optimized for expression in human cells. The vectors have a unique *Not *I site between the scs' insulator and the reef fluorescent protein marker gene for insertion of the gene of interest.

The fourth scs/scs' vector we have constructed (Figure 2D) has the *D. melanogaster cinnabar *(*cn*) eye color marker gene that encodes the enzyme kynurenine 3-monooxygenase [28]. The *cn *gene has been used for selection of transgenic *Ae. aegypti *[29] but potentially could be used in other insect species where mutations in the corresponding kynurenine 3-monooxygenase gene have been identified. The vector has a unique *Pac *I site downstream from the *cn *polyadenylation site that could be used for future gene cloning. Although all scs/scs' vectors contain only a single cloning site, both *Not *I and *Pac *I have 8 bp recognition sequences and so would be unlikely to cut most gene constructs.

All vectors have been successfully used for germ-line transformation of *D. melanogaster*. Three lines were obtained with the DsRed marker, five with AmCyan, six with ZsGreen and two with the *cn *gene. Lines carrying the GMR-3xP3-reef fluorescent protein marker genes were easily identified as the eyes were brightly fluorescent (Figure 2). This suggests that the GMR-3xP3 promoter is a strong driver of gene expression and that the ZsGreen and AmCyan fluorescent proteins can be used as markers in insects. With the cyan filter set significant levels of ZsGreen expression were observed (Figure 2D). Thus this filter set could not be used to separately observe ZsGreen and AmCyan expression in the same tissue. The frequency of transformation (5–10% of G_0 _crosses) was comparable to what we obtained previously with non-insulated *piggyBac *vectors [23].

**Figure 3:**
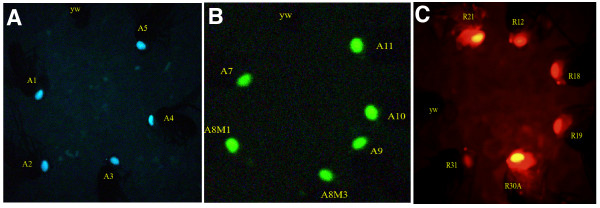
**Comparison of fluorescence intensities of lines carrying insulated and uninsulated marker genes**. A representative fly is shown from each of the lines carrying the insulated GMR-3xP3-AmCyan (A) and GMR-3xP3-ZsGreen (B) marker genes. Little variation in fluorescence intensity is seen between lines suggesting position effects had been minimized by the flanking scs and scs' insulator elements. In contrast lines carrying an uninsulated 3xP3-DsRed1 marker gene (C) show considerable variation in fluorescence intensity. Some lines are barely detectable above background fluorescence (*y w*) while other lines show very strong fluorescence.

By visual inspection the fluorescence or color intensity appeared to be similar between lines carrying the same marker gene. A representative fly from each of the five AmCyan and six ZsGreen lines are shown in Figure 3, along with the *y w *parental strain. The low level of variation in marker gene expression between lines is in contrast to that previously reported for lines carrying the uninsulated 3xP3-EGFP, 3xP3-EYFP and 3xP3-ECFP marker genes [30,31]. In a recent experiment six transformed lines were obtained with a *piggyBac *vector containing the uninsulated 3xP3-DsRed1 marker gene described previously [22]. These lines also show considerable line-to-line variation in fluorescence intensity (Figure 3C). These observations suggested that the scs/scs' insulators are minimizing position effects.

To confirm these visual observations, more quantitative analyses were performed on the expression levels of the insulated AmCyan and ZsGreen lines and uninsulated DsRed1 lines shown in Figure 3. Two methods were used to estimate fluorescence intensities. Firstly, flies heterozygous for a marker gene were selected at random and examined under a stereo fluorescence microscope. Images were digitally captured and analyzed using software that can quantify color intensity of a selected region of the image (Table 1). All AmCyan, ZsGreen and DsRed1 lines were more significantly more intense than the control *y w *parental strain (one way analysis of variance, P < 0.05). Confirming visual observations, all AmCyan and most of the ZsGreen lines were not significantly more intense than other lines carrying the same marker gene. The two brightest ZsGreen lines (A10, A11) were significantly more intense than the three weakest lines (A7, A8M1, A9). However, the difference in color intensity between the strongest and weakest lines was less than 1.5 fold. In contrast, pairwise comparisons of the uninsulated 3xP3-DsRed1 lines found that the brightest line (R30A) was significantly more intense than all other lines as was the second brightest line (R21). Further, the brightest line had more than 20 times the color intensity of the weakest line.

In the second method, the fluorescence intensities of soluble protein extracts prepared from homogenized heads were measured using a fluorometer (Table 2). In general the results are in agreement with those obtained with the first method although there was more variability between lines, which was reflected in the statistical analyses. All AmCyan and ZsGreen lines had significantly higher fluorescence levels than the control strain (one way analysis of variance, P < 0.05). In pairwise comparisons the AmCyan lines were not significantly different from each other with the exception that the two brightest lines (A4, A5) had significantly more fluorescence than the weakest line (A2). The brightest two ZsGreen lines (A11, A8M3) had significantly higher levels of fluorescence than the other four ZsGreen lines. Other pairwise comparisons found little significant differences except that the A9 and A10 lines had significantly higher fluorescence levels than the A7 and A8M1 lines. While a statistically significant difference was found in some pairwise comparisons it is important to note that at most the fluorescence intensities differ by no more than two fold. While insulators do protect against position effects, it would be expected that the expression levels of insulated transgenes would not be identical between lines [13,32]. In contrast the fluorescence levels of the uninsulated 3xP3-DsRed1 lines differ by as much as 7 fold (Table 2). In pairwise comparisons, the brighter lines (R30A, R21) had significantly more fluorescence than all the other lines carrying the same marker gene.

### *piggyBac *vectors containing the *gypsy *or β-*globin *HS4 insulators

**Figure 4:**
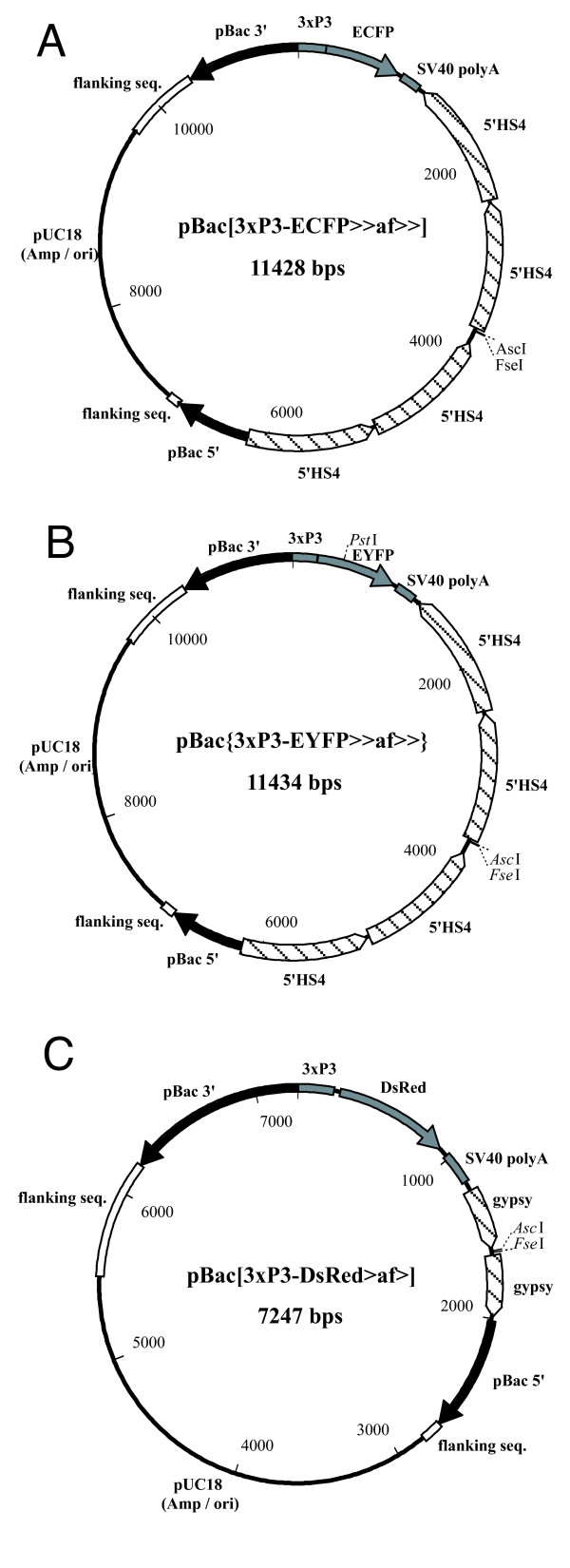
***piggyBac *vectors containing the β-*globin *5' HS4 and *gypsy *insulator elements**. The vectors contain either the ECFP (A), EYFP (B) or DsRed1 (C) marker genes. The β-*globin *5' HS4 (A, B) and *gypsy *(C) insulator elements do not bracket the marker genes but do flank the unique *Asc*I and *Fse*I cloning sites. The vectors have 685 bp of 5' terminal *piggyBac *sequence and 1051 bp of 3' terminal sequence.

The β-*globin *5' HS4 insulator sequences were introduced into 3xP3-EYFP and 3xP3-ECFP marked *piggyBac *constructs. The resulting vectors, pBac{3xP3-EYFP>>af>>} and pBac{3xP3-ECFP>>af>>} were used to insulate transgene constructs of the tTA/*TRE *binary expression system when introducing conditional embryonic lethality into *Drosophila melanogaster *[13]. For completeness, maps of these vectors are shown in Figure 4. The transgenic lines carrying the insulated *TRE*-responder constructs seemed to work more reliably and to mediate higher gene expression than their non-insulated counterparts [13], which indicates the functionality of the insulator elements in protecting the embedded transgenes from position effects.

The chicken β-*globin *5' HS4 insulator sequences are relatively long (two times 2.4 kb) and seem to work efficiently. However, the tandem repetition of the element on either side of the insulated region causes problems of recombination when growing these plasmid constructs in bacteria. These constructs can only be grown in recombinase-negative bacteria, like STBL2 (Life Technologies, Rockville, MD). To avoid this complication in generating insulated constructs, another smaller insulator from the *gypsy *element was chosen and introduced into a 3xP3-DsRed marked *piggyBac *construct [22] resulting in the transformation vector pBac{3xP3-DsRed>af>} (Figure 4C). This vector has been successfully used in several germ-line transformation experiments in *D. melanogaster*. More than thirty lines have been generated at an efficiency comparable to that reported previously for *piggyBac *vectors carrying 3xP3-fluorescent protein marker genes [31].

The *gypsy *and chicken β-*globin *5' HS4 vectors contain single *Asc*I and *Fse*I endonuclease restriction sites, which allow for a described two step cloning procedure using the shuttle vector pSLfa1180fa [31]. In contrast to the scs/scs' vectors, the *gypsy *or chicken β-*globin *5' HS4 vectors were constructed to insulate only the transgene of interest without insulating the transformation marker gene. In this arrangement, the transgene is also insulated from the *cis*-regulatory sequences driving marker gene expression.

## Conclusion

We have described insulated *piggyBac *vectors carrying different fluorescent protein and eye color markers that have been successfully used for germ-line transformation in *D. melanogaster*. The use of the reef coral fluorescent proteins AmCyan and ZsGreen is the first report that these proteins can serve as effective transformation markers in insects. It is likely that all of the insulated vectors will provide some protection against position effects in most insect species. The chicken β-*globin *HS4 insulator is of vertebrate origin yet effectively blocks position effects in *D. melanogaster *[17]. Although the *gypsy *and scs insulators are from *D. melanogaster *they have both been shown to be effective at protecting against heterochromatin silencing in a cultured human cell line [33]. Thus the vectors described herein should facilitate fine control of gene expression in a broad range of insect species. Also, these insulated vectors may be modified by changing the respective promoter to use them in mammalian systems.

## Methods

### Recombinant DNA

pB [SCS-GMR-3xP3-DsRed-SCS']: The *piggyBac *plasmid p3E1.2 (provided by M. Fraser) was first modified by the removal of the *Sal*I and *Eco*RI sites. This modified 3E1.2 was then digested with *Pst*I and *Bgl*II, blunt ended with T4 DNA polymerase, and the scs-scs' cassette from pELBA6 (provided by Paul Schedl) inserted to create pB [SCS-SCS']. The plasmid pB [SCS-SCS'] was digested with *Sal*I and a *Xho*I-*Sca*I fragment containing the GMR enhancer [24] inserted to create pB [SCS-GMR-SCS']. The 3xP3 fragment from pHer [3xP3-EGFPaf] [31] was inserted into pDsRed (BD Biosciences Clontech) to create p3xP3DsRed. Subsequently, a fragment containing the polyadenylation site from the *D. melanogaster hsp70 *gene was cloned into the *Not*I site of p3xP3DsRed to create p3xP3DsRedpolyA. Finally, the 3xP3DsRedpolyA fragment was cloned into pB [SCS-GMR-SCS'] to create pB [SCS-GMR-3xP3-DsRed-SCS'].

pB [SCS-GMR-3xP3-AmCyan-SCS'] and pB [SCS-GMR-3xP3-ZsGreen-SCS']: The plasmid pBCpolyA was created by cloning the *hsp70 *3' flanking (polyA) sequence as a *Stu*I-*Pst*I fragment into the *Eco*RV-*Pst*I sites of pBCKS+ (Stratagene). Fragments containing the AmCyan and ZsGreen genes (BD Biosciences Clontech) were then cloned into the *PspOM*I-*Asp*718 sites of pBCpolyA as *Not*I-*Asp*718 fragments to create pBCAmCyanpolyA and pBCZsGreenpolyA respectively. pB [SCS-GMR-3xP3-DsRed-SCS'] was then digested with *Not*I-*Asp*718, the DsRedHsp70polyA gene cassette removed and substituted with the *Not*I-*Asp*718 fragments containing either the AmCyanpolyA or the ZsGreenpolyA sequences to create pB [SCS-GMR-3xP3-AmCyan-SCS'] and pB [SCS-GMR-3xP3-ZsGreen-SCS'] respectively.

pB [SCS-*cn*-SCS']: A 7.2 kb *Asp*718/*Sph*I fragment containing the *D. melanogaster cn *gene [28] was treated with T4 DNA polymerase to make blunt ends then inserted into the *Eco*RV site of pElba6 (kindly provided by Paul Schedl), which contains the scs and scs' insulators. The resulting plasmid was digested with *Not*I and *Bsp*120I and ligated with *Not*I digested pB [Ccw] (provided by A. Handler) essentially replacing the *C. capitata w *gene with the scs-*cn*-scs' cassette.

The construction of the β-*globin *5' HS4 insulator vectors, pBac{3xP3-EYFP>>af>>} and pBac{3xP3-ECFP>>af>>}, is described in [13]. pBac{3xP3-DsRed>af>} was constructed by introducing a 400 bp PCR fragment, amplified with primers NH_FgyFse (5'-CCGGCCGGCCTTGAATTCGAGCTCGGTACC-3') and NH_RgyNgo (5'-CCCGCCGGCAATTGATCGGCTAAATGG-3') from pBS-*gypsy*HIII (provided by V. Pirrotta) and cut with *Ngo*MIV, into *Fse*I-cut pBac{3xP3-DsRed>af}, which itself was constructed by introducing a 400 bp PCR fragment, amplified with primers NH_FgyBss (5'-CCCGCGCGCTTGAATTCGAGCTCGGTACC-3') and NH_RgyAsc (5'-CCGGCGCGCCAATTGATCGGCTAAATGG-3') from pBS-gypsyHIII and cut with *Bss*HII, into *Asc*I-cut pBac{3xP3-DsRedaf} [22]. During the ligations in the different steps, the *Asc*I site is restored at the 3'-end of the first *gypsy *element and the *Fse*I site is restored at the 5'-end of the second *gypsy *element, respectively. In this construct, ">" symbolizes the insulator region of the *gypsy *retrotransposon.

### Transformation of *D. melanogaster *and detection of fluorescent proteins

Germ-line transformation of *D. melanogaster *was carried out essentially as previously described [34]. The recipient strains used were *y w *and *cn bw *for *piggyBac *vectors containing the fluorescent protein and *cn *marker genes respectively. Transformants carrying the GMR-3xP3-reef fluorescent proteins marker genes were identified by florescence using an Olympus SZX12-RFL3 dissecting microscope with the appropriate excitation/barrier filter sets (EX 420–460 nm, EM 480–520 nm for AmCyan; EX460-90 nm, EM510-550 nm for ZsGreen; EX520-550 nm, EM580+ nm for DsRed). Images were captured using an Olympus DP70 digital camera. To compare expression levels of fluorescent proteins, single flies were selected at random from each line, anaesthetized then examined together. Thus identical camera settings were used for flies from each line. The intensity of fluorescence in the eyes was measured using the analySIS software package (Soft Imaging System GmbH) from an arbitrary selection of eye ommatidia using appropriate filter sets for the fluorescent protein marker. The DP-70 camera settings were exposure time 500 microseconds for AmCyan and ZsGreen lines and 2500 microseconds for the uninsulated DsRed1 lines. Further there was no color offset and sharpness and contrast were at maximum. These settings were used to avoid pixel saturation, while still detecting the weakest lines. Flies heterozygous for the marker gene were obtained by back crossing to the parental *y w *strain. The amount of fluorescence in the eyes of the transgenic lines was also quantified using a fluorometer. Heads of ten flies heterozygous for the fluorescent marker were dissected, snap frozen in liquid nitrogen and ground in 100 μL RIPA buffer (50 mM Tris HCl, pH 8.5; 300 mM NaCl; 1× protease inhibitors [Roche]), kept on ice for 30 minutes and centrifuged for 10 minutes at 14,000 rpm at 4°C using an Eppendorf 5417R microcentrifuge and the supernatant collected. 15–60 μL aliquots were taken and fluorescence detected using a Fluorostar Galaxy fluorometer (BMG Technologies) at the appropriate wavelengths (AmCyan: EX450 nm, EM490 nm/10 nm; ZsGreen EX450 nm, EM500 nm/12 nm; DsRed1, EX450 nm, EM590 nm/20 nm). For all extracts there was a linear response between volume of extract and level of fluorescence. Three extractions were performed for each line. The amount of protein in the supernatant was estimated using the Bradford assay at 595 nm. Statistical analyses were performed using the SigmaStat for Windows v3.11 software package. The Holm-Sidak method was used for the one way analysis of variance calculation.

## Authors' contributions

AS, constructed the scs/scs' AmCyan and ZsGreen vectors, performed assays for quantifying fluorescence intensities, assisted with transgenesis and drafting of manuscript.

AA, constructed the scs/scs' DsRed vectors and assisted with transgenesis.

EJB, performed transgenesis experiments with AmCyan and ZsGreen vectors.

JCH, constructed scs/scs' vector with cinnabar marker gene

XL, performed transgenesis experiments with scs/scs' vectors containing DsRed and *cn *marker genes.

CH, constructed *gypsy *and β-*globin *insulated vectors and performed transgenesis experiments with these vectors.

EAW, supervised development of *gypsy *and β-*globin *insulated vectors and assisted with drafting of manuscript.

MJS, designed the scs/scs' vectors, assisted with screening for transgenics, drafted the manuscript and supervised Massey personnel.

## Figures and Tables

**Table 1:** Fluorescence intensity in the eyes of transgenic lines expressing reef coral fluorescent marker proteins. Live anaesthetized flies were observed with an Olympus SZX12-RFL3 microscope, image captured with an Olympus DP70 digital camera and color intensity mesured using the analySIS software package. Brief exposure times were used to avoid pixel saturation.

Line	Marker^a^	Insulated^b^	Mean^c^	SD
A1	GMR-3xP3-AmCyan	Yes	123.9	13.9
A2	GMR-3xP3-AmCyan	Yes	125.3	9.4
A3	GMR-3xP3-AmCyan	Yes	128.6	11.8
A4	GMR-3xP3-AmCyan	Yes	137.6	9.6
A5	GMR-3xP3-AmCyan	Yes	141.3	16.0
*y w*^*d*^	None		23.5	2.4
				
A7	GMR-3xP3-ZsGreen	Yes	75.8	9.5
A8M1	GMR-3xP3-ZsGreen	Yes	82.0	7.8
A8M3	GMR-3xP3-ZsGreen	Yes	93.3	3.2
A9	GMR-3xP3-ZsGreen	Yes	79.5	6.2
A10	GMR-3xP3-ZsGreen	Yes	100.8	4.8
A11	GMR-3xP3-ZsGreen	Yes	101.2	7.1
*y w*	None		17.6	5.2
				
R31	3xP3-DsRed1	No	20.6	2.4
R30A	3xP3-DsRed1	No	246.7	4.4
R19	3xP3-DsRed1	No	37.5	5.2
R18	3xP3-DsRed1	No	29.9	7.0
R12	3xP3-DsRed1	No	38.5	6.2
R21	3xP3-DsRed1	No	115.6	4.7
*y w*	None		10.5	0.4

^a^All lines heterozygous for marker gene.

^b^scs and scs' insulators bracket the GMR-3xP3 marker genes.

^c^Average of three individuals in arbitrary units

^d^Parental stock used in all germ-line transformation experiments

**Table 2:** Fluorescence intensities of soluble protein extracts prepared from heads of transgenic lines expressing reef coral fluorescent proteins.

Line	Marker^a^	Insulated^b^	Mean Fluorescence/μg protein^c^	SD
A1	GMR-3xP3-AmCyan	Yes	857	173
A2	GMR-3xP3-AmCyan	Yes	629	69
A3	GMR-3xP3-AmCyan	Yes	846	88
A4	GMR-3xP3-AmCyan	Yes	1065	257
A5	GMR-3xP3-AmCyan	Yes	1016	83
*y w*^*d*^	None		13	37
				
A7	GMR-3xP3-ZsGreen	Yes	758	71
A8M1	GMR-3xP3-ZsGreen	Yes	828	41
A8M3	GMR-3xP3-ZsGreen	Yes	1558	20
A9	GMR-3xP3-ZsGreen	Yes	1205	176
A10	GMR-3xP3-ZsGreen	Yes	1213	185
A11	GMR-3xP3-ZsGreen	Yes	1737	82
*y w*	None		41	43
				
R31	3xP3-DsRed1	No	118	24
R30A	3xP3-DsRed1	No	859	56
R19	3xP3-DsRed1	No	215	135
R18	3xP3-DsRed1	No	169	51
R12	3xP3-DsRed1	No	171	31
R21	3xP3-DsRed1	No	342	14
*y w*	None		122	43

^a^All lines heterozygous for marker gene

^b^scs and scs' insulators bracket the GMR-3xP3 marker genes.

^c^Average of three protein extracts each prepared from ten homogenized heads from each line. Fluorescence in arbitrary units.

^d^Parental stock used in all germ-line transformation experiments
